# Water-Soluble Ruthenium (II) Complex Derived From Optically Pure Limonene and Its Microencapsulation Are Efficient Tools Against Bacterial Food Pathogen Biofilms: *Escherichia coli*, *Staphylococcus aureus*, *Enteroccocus faecalis*, and *Listeria monocytogenes*

**DOI:** 10.3389/fmicb.2021.711326

**Published:** 2021-11-15

**Authors:** Simon Khelissa, Yousra El Fannassi, Samah Mechmechani, Sakhr Alhuthali, Mohamed Amin El Amrani, Adem Gharsallaoui, Alexandre Barras, Nour-Eddine Chihib

**Affiliations:** ^1^Univ. Lille, CNRS, INRAE, Centrale Lille, UMR 8207 - UMET - Unité Matériaux et Transformations, Lille, France; ^2^Université Abdelmalek Essaadi, Faculté des Sciences, Laboratoire de Chimie Organique Appliquée, Tétouan, Morocco; ^3^Department of Chemical Engineering, Imperial College London, London, United Kingdom; ^4^Univ Lyon, Université Claude Bernard Lyon 1, CNRS, LAGEPP UMR 5007, Villeurbanne, France; ^5^Univ. Lille, CNRS, Centrale Lille, Univ. Polytechnique Hauts-de-France, UMR 8520-IEMN, Lille, France

**Keywords:** ruthenium (II), limonene, complexation, microencapsulation, biofilm, antibacterial agent, food pathogen, biofilm removal

## Abstract

Bioactive aminooxime ligands based on optically pure (R)-limonene have been synthesized in two steps. Their ruthenium (II) cationic water-soluble complex was prepared by a reaction between dichloro (para-cymene) ruthenium (II) dimers and aminooxime ligands in a 1:2 molar ratio. Antibacterial and antibiofilm activities of the synthetized complex were assessed against *Escherichia coli*, *Staphylococcus aureus*, *Listeria monocytogenes*, and *Enterococcus faecalis.* The results revealed that the ruthenium (II) complex has higher antibacterial and antibiofilm activities in comparison with free ligands or the enantiopure (R)-limonene. Moreover, microencapsulation of this complex reduced its cytotoxicity and improved their minimum inhibitory concentration and antibiofilm activity toward the considered bacteria. The ruthenium (II) complex targets the bacterial cell membrane, which leads to rapid leakage of intracellular potassium. Our study suggests that the developed ruthenium (II) complexes could be useful as an alternative to conventional disinfectants.

## Introduction

Unit operation hygiene is a major challenge for healthcare establishments and agro-food industries as the surface contamination can lead to infectious diseases and food poisoning (Abdallah et al., 2014). Despite the implementation of preventive measures such as frequent cleaning and disinfection procedures, persistent bacteria can form biofilms on abiotic surfaces if the growth conditions are suitable. Biofilms allow bacteria to increase resistance to environmental stresses, cleaning, and disinfection treatments compared with planktonic form bacteria (Bridier et al., 2011; Khelissa et al., 2017, 2018, 2019; Singh et al., 2017; Cadena et al., 2019).

Biosourced antimicrobials represent a promising alternative to chemically synthesized antimicrobials, such as quaternary ammonium compounds. Terpenes from aromatic plant essential oils show a good antibacterial activity (Guimarães et al., 2019). However, there are many challenges to be used as antimicrobial faces, such as high volatility, non-water miscibility, and cytotoxicity (Fisher and Phillips, 2008; Mahizan et al., 2019). Mediterranean countries produce high amounts of citrus fruits and aromatic plants. The valuation of citrus byproducts, as a source of renewable and biodegradable raw materials, through the production of essential oils, constitutes a major challenge for sustainable development. The chemical composition of the essential oil extracted from the peel of citrus fruits mainly contains low-priced enantiopure limonene (80–95%) as well as other terpenoids and aromatic compounds (Bourgou et al., 2012).

Limonene has many applications related to its antimicrobial activity (Bomgardner, 2011; Ciriminna et al., 2014). The degree of toxicity of limonene depends on its level of oxidation. Indeed, several studies have shown that oxidation derivatives of limonene exhibit greater antibacterial toxicity than limonene (Chubukov et al., 2015). Terpene (abundant agro-resource) use can be an effective tool to reduce the microbiological risk related to contamination of the surfaces of materials in contact with food-based products. Furthermore, it has been reported that the compounds containing amine and oxime functions in their structures have good antibacterial activities (Sahyoun et al., 2019), as well as the complexes of various functionalized ligands. It has been also reported that the antibacterial activity of some ligands and their associated metal complexes show higher activity than free ligands against bacteria (Samota and Seth, 2010). In addition, it has been reported that some metal complexes are water soluble (Bayón Castañón et al., 2018). This characteristic constitutes an advantage for their use over the use of terpenes as antibacterial compound.

Bacterial biofilms are encased in a self-produced matrix composed of extracellular polymeric substances (EPS) such as polysaccharides, proteins, lipids, and extracellular DNA. The biofilm matrix represents a diffusion barrier, which can delay or prevent the interaction of biocides with microbial cells. Thus, the interactions between the EPS and biocides can lead to biocide sequestration or repulsion with a significant decrease in the bioactive concentration of the used biocides (Pereira and Vieira, 2001). The encapsulation of biocides could prevent the interactions between biocides and the EPS of the biofilm matrix. This can enhance the effectiveness of the antimicrobials. In addition, microencapsulation could lead to the reduction of the amount of biocide used and minimize their environmental impact. The microencapsulation of biosourced terpenes and their derivative complexes as biocides could be also an efficient tool to overcome their high volatility, non-water miscibility, and cytotoxicity.

In this context, the aim of this work was to assess the antibacterial and the antibiofilm activities of a chiral aminooxime ligand L3 [(1S, 4R)-1-benzylamino-p-menth-8-en-2-one oxime] obtained by the functionalization of R-limonene and its water-soluble ruthenium complex. In addition, the microencapsulation was used as an innovative tool to enhance the antimicrobial and the antibiofilm activities of the limonene and its derivative and to reduce the amount of the biocide used in order to set up efficient and environmentally friendly disinfection procedures for closed and open food contact surfaces.

## Materials and Methods

### Synthesis of the Aminooxime Ligand L3 and the RuL3 Complex

The synthesis of the aminooxime ligand L3 and the RuL3 complex were performed according to the procedures described in the literature (Ibn El Alami et al., 2012; Benabdelouahab et al., 2015; El Alami et al., 2015).

### Ligand (1S,4R)-1-Benzylamino-p-Menth-8-en-2-One Oxime Ligand: L3

Isopentyl nitrite (isoamyl nitrite) (CH_3_)_2_CH(CH_2_)_3_ONO and concentrated hydrochloric acid (37%) were added to (R)- or (S)-limonene at −5°C. The reaction mixture was stirred at the same temperature for 1 h. The precipitate formed was washed with cold methanol and dried to obtain a solid corresponding to the nitrosochloride A of (R)- or (S)-limonene (yield = 47%). A mixture consisting of nitrosochloride A (13 g, 32.5 mmol) and benzylamine (13 ml, 119.6 mmol) in ethanol (20 ml) was heated until a clear solution was obtained. This solution was cooled to −5°C, and hydrochloric acid (37%) was added dropwise (up to acid pH). The white obtained solid, corresponding to L3 hydrochloride, was washed successively with ethanol and diethyl ether, then basified with triethylamine. The solution was recovered with diethyl ether, washed with water, and dried on MgSO_4_. Evaporation of the solvent provides the final aminooxime L3 as a white solid (7 g, yield = 71%).

*Elemental analysis (%):* C_1__7_H_2__4_N_2_O
Calculated: C, 74.96; H, 8.88; N, 10.28.
Found: C, 74.59; H, 9.064; N, 10.17.

### Complex {RuCl[(Para-Cymene)][Aminooxime L3]}+Cl^–^: RuL3

(1S,4R)-Benzylaminooxime L3 (0.133 g; 0.48 mmol) and [RuCl_2_(p-cymene)]_2_ (0.15 g; 0.24 mmol) were introduced into a Schlenk tube and solubilized in anhydrous dichloromethane (7 ml). The reaction mixture was stirred for 30 min at room temperature. Diethylether (4.5 ml) was added dropwise, and the mixture was maintained at −5°C overnight. After filtration, the residue was dried under reduced pressure to give RuL3 complex as a yellow powder (0.26 g, yield = 81%).

*Elemental analysis (%):* RuC_2__7_H_3__8_N_2_OCl_2_
Calculated: C, 56.05; H, 6.62; N, 4.84.
Found: C, 55.65; H, 6.90; N, 4.77.

### Bacterial Strains

*Staphylococcus aureus* CIP 4.83, *Escherichia coli* CIP 54127, *Enterococcus faecalis* isolated from French cheese, and *Listeria monocytogenes* ATCC 35152 were used in this study. Strains were maintained at −20°C in Tryptic Soy Broth (TSB; Biokar Diagnostics, Pantin, France), supplemented with 40% (v/v) glycerol. For preculture preparation, each strain was grown in 5 ml of TSB for 24 h at 37°C. The main cultures were started by inoculating 10^4^ CFU/ml from the preculture in 50 ml. Bacterial cultures were incubated overnight at 37°C, under continuous shaking (160 rpm).

### Determination of Minimum Inhibitory Concentrations

The microdilution method was used to determine the minimum inhibitory concentration (MIC). Limonene was dissolved in TSB supplemented with 2% (v/v) dimethyl sulfoxide (DMSO). Twofold serial dilutions of each tested compound was prepared in a Bioscreen well microtiter plate over the range of 0–100 mg/ml (in TSB). Bacterial suspensions were adjusted to 10^6^ CFU/ml. In addition, wells containing each strain inoculum in TSB without the tested component were measured as positive control, DMSO as vehicle control, and only TSB as negative control. The microdilution plates were incubated at 37°C under continuous shaking. The optic density (OD) at 600 nm was measured every 2 h for 24 h. The MIC was defined as the lowest concentration that prevented growth as measured by optical density. All experiments were done in triplicate using different microplates.

### Antibiofilm Assays

Bacterial cells were harvested from overnight cultures by centrifugation (5,000 × g, 5 min, 20°C) and washed twice with potassium phosphate buffer (PPB; 100 mM, pH 7). Bacterial suspensions were adjusted to 10^7^ CFU/ml in PPB. Three milliliters of suspension cells was deposed on sterile stainless steel (SS) slides. After 1 h of static incubation at 20°C, SS slides were rinsed with 5 ml of PPB then covered with 3 ml of TSB before incubation for 24 h at 37°C to allow biofilm formation on SS. Biofilms were rinsed with PPB then treated with the different considered compounds (at the MIC) for 10 min. Treated biofilms were immersed in 20 ml of neutralizing solution (Toté et al., 2010) containing 1 g of 1-mm diameter glass beads in 100-ml sterile pots. Pots were vortexed for 30 s followed by sonication for 5 min (37 kHz, 20°C) (Elmasonic S60H, Elma, Germany). Tenfold serial dilutions were made in PPB. Samples of 100 μl were spread onto TSA plates and incubated at 37°C for 24 h. The colony number was counted, and results were expressed in log CFU/cm^2^. PPB was used as negative control. The results represent the means of three independent experiments, and in each experiment three slides were used.

### Scanning Electron Microscope Observation

The inner structures of RuL3 microcapsules were investigated by scanning electron microscope (SEM-JEOL-JSM-7800FLV, Japan). The powder containing RuL3 microcapsules was fractured by moving a razor blade perpendicularly through a layer of microcapsules. The morphology of bacterial cells after treatment with different compounds was assessed by SEM. One-milliliter volume of treated and untreated cells was filtered through a 0.2-μm polycarbonate membrane filter (Schleicher and Schuell, Dassel, Germany) then fixed at 4°C for 4 h in 2% glutaraldehyde in cacodylate buffer 0.1 M pH 7. Fixed samples were then dehydrated in an ascending ethanol series (50, 70, 95, and 2 × 100% (v/v) ethanol) for 15 min at each concentration. Samples were critical point dried and coated with thin carbon film before examination in the SEM. Microscopy was performed with a JEOL JSM-7800F LV microscope at 3 kV.

### Epifluorescence Microscopy Observation

Biofilms were treated with microencapsulated RuL3. For control condition, biofilms were treated with PPB. Biofilms were stained with LIVE/DEAD BacLight kit (Invitrogen Molecular Probes, United States), according to the instruction of the manufacturer for 15 min, then washed by gently dipping in sterile distillated water, air dried in the dark before being observed under an epifluorescence microscope (Olympus BX43, Germany). The green cells were considered viable, and the red ones were defined as non-viable.

### Cytotoxicity Assay

The HeLa cell line (ATCC^®^ CCL-2™, ECACC, Sigma Aldrich, Saint-Quentin Fallavier, France) was cultured and maintained in Dulbecco’s modified Eagle’s medium (DMEM, Gibco^®^) supplemented with 10% fetal bovine serum (FBS, Gibco^®^) and 1% penicillin–streptomycin (Gibco^®^) in a humidified incubator at 37°C and 5% CO_2_. Cells were seeded at a density of 10^4^ cells/well in a 96-well plate and grown for 24 h before assay. The culture medium was replaced with a fresh medium that contains the compounds from 12.5 to 800 μg/ml. After 24 h, the old medium was removed, and cells were washed with PBS. The cell viability was evaluated using the resazurin cell viability method. Briefly, 100 μl of the resazurin solution (11 μg/mL) in complete medium was added to each well, and the plate was incubated for 4 h in a humidified incubator. The fluorescence emission of each well was measured at 593 nm (20-nm bandwidth) with an excitation at 554 nm (18-nm bandwidth) using a Cytation™ 5 Cell Imaging Multi-Mode Reader (BioTek Instruments SAS, France). Each condition was replicated three times, and the mean fluorescence value of non-exposed cells was taken as 100% cellular viability.

### Ruthenium Complex-Induced Cell Membrane Permeabilization

*E. coli* grown overnight at 37°C cells was concentrated to 10^10^ CFU/ml (5,000 × g, 15 min, 20°C). The concentrated bacterial suspension was mixed with free or microencapsulated RuL3 (prepared in 50 mM MOPS buffer at MIC as final concentration) or TSB, prepared in 50 mM MOPS buffer (negative control). The K^+^ concentration at time zero was measured in a 10-fold dilution of the bacterial suspension filtrate (0.2 μm, Sartorius™ Minisart™ NML Syringe Filters, France) before contact with different solutions. Tested compounds were introduced to cell suspension after 20 min. Samples were filter sterilized at 10, 20, 30, 40, 50, 70, and 90 min. Each sample was removed using a sterile plastic syringe attached to a sterile needle to enable easy access to the reaction mixture suspension through a silicon cap. The K^+^ concentration in filtrate samples was determined using a Varian SpectrAA 55/B atomic absorption spectrometer in flame emission mode (wavelength 766.5 nm; slit 0.7-nm high; air–acetylene flame).

### Extracellular GFP Intensity Assessment

The extracellular GFP intensity assessment was carried out according to Khelissa et al. (2021b). An overnight culture (37°C) of *Escherichia coli* GFP (ATCC 25922GFP) in TSB supplemented with 100 mg/ml of ampicillin was used. After centrifugation (5,000 × *g*, 5 min, 20°C), pelleted cells were washed with HEPES buffer (5 mM, pH 7.2). A 5 ml of concentrated bacterial cell suspensions (1,010 CFU/ml) was added to 45 ml of free DTAC, free or encapsulated RUL3 prepared in HEPES buffer (final concentration corresponding to the MIC in a final volume of 50 ml) or HEPES buffer as a negative control. At 0, 5, and 10 min before cells were exposed to DTAC, free or encapsulated RUL3, 10-fold dilutions of the concentrated inoculum were made in HEPES buffer, then filter sterilized (Sartorius™ Minisart™ NML 0.2-ml Syringe Filters, France). These filtrates were used to assess the extracellular GFP fluorescence intensity before the antibacterial treatment. Samples were filter sterilized at 5, 10, 15, 20, and 30 min after the addition of bacterial cells to the reaction vessel containing the antibacterial solution. Aliquots (200 ml) of the filtered supernatant samples were transferred to a 96-well microplate, and GFP fluorescence was quantified using a BioTek fluorescence spectrophotometer (BioTek Instruments SAS, France) with excitation at 485 nm and emission at 510 nm. The fluorescence intensity ratio of samples to HEPES buffer was plotted vs. contact time. The results represent the average of three independent experiments.

### Zeta Potential Measurement

The electric charges (ζ-potential) of RuL3 at different pH values were determined using a Zetasizer Nano ZS90 (Malvern Instruments, Malvern, United Kingdom). The samples were diluted with imidazole acetate buffer adjusted to the adequate pH. The mean ζ-potential (ZP) values [± SD (standard deviation)] were obtained from the instrument. The measurements were repeated three times for each suspension.

### Microencapsulation of RuL3 by Spray Drying

A stock maltodextrin solution was added to an aqueous solution of RuL3 in order to have a final composition (w/w) of 20% maltodextrin DE 19 and 0.4% RuL3 in imidazole/acetate buffer (5 mM, pH 7). Maltodextrin DE 19 (dextrose equivalent value of 19) were obtained from Roquette-freres SA (Lestrem, France). The mixture was stirred for 30 min and then spray dried using a laboratory scale device equipped with a 0.5-nm nozzle atomizer (Mini Spray-Dryer Büchi B-290, Switzerland). The operational conditions of the drying process were feed flow rate 0.5 L/h, inlet air temperature 180 ± 2°C, and outlet air temperature 80 ± 5°C. After spray drying, the powder was collected in sealed containers and stored at 4°C until microbiological tests. Spray-dried microcapsules without RuL3 (empty capsules) were used as control.

### Statistical Analysis

All experiments were carried out at least three times. Statistical analysis was performed with IBM SPSS 19 statistics software following one-way ANOVA. Results were considered significantly different when *p* < 0.05.

## Results

### Zeta Potential of RuL3 as a Function of pH

**FIGURE 1:**
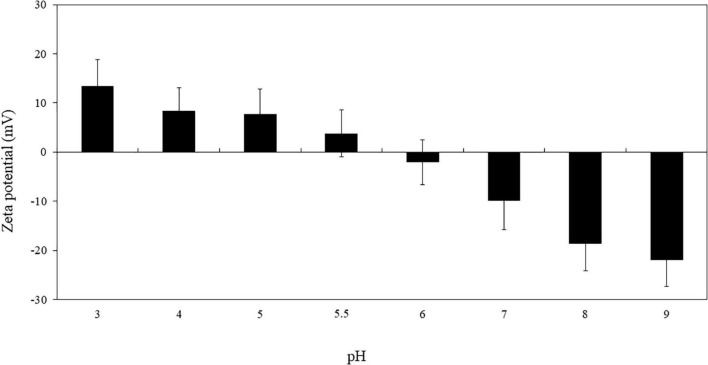
Zeta potential of RuL3 as a function of pH (imidazole/acetate buffer, 5 mM). Data are presented as means (± SD) of three independent repextineats in triplicate.

To assess the charge of the RuL3 in our conditions of bacterial cells and biofilm treatments, the zeta potential of RuL3 as a function of pH was measured as shown in Figure 1. The results show that the zeta potential of RuL3 is largely pH dependent and that the charge of the molecule changes from positive to negative as the pH increases. The point at which the overall charge of the molecule is neutral was between 5.5 and 6 (Figure 1).

### Morphology and Inner Structure of Spray-Dried Microcapsules

**FIGURE 2:**
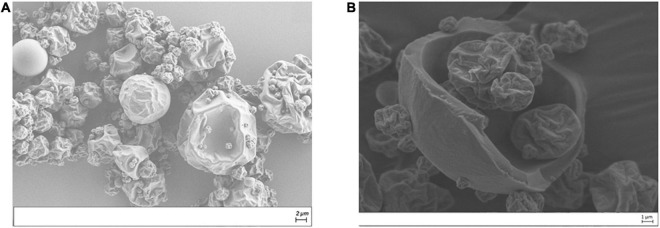
Scanning electron microscope (SEM) micrographs of spray-dried solution containing 1 g of RuL3 and 50 g of maltodextrin DE 19 in imidazole acetate buffer at pH 7 [external **(A)** and internal **(B)** structures].

Figure 2 shows the scanning electron micrographs of microcapsules obtained by spray drying the RuL3 solutions in the presence of maltodextrin DE19. The microcapsules consisted of well-separated spherical particles, having heterogeneous diameters, rounded shape, and generally dented surfaces. In fact, the viscosity of feed solutions was relatively high, and consequently, the particles were not well formed during the drying process. They tend to stick to each other and were not very well separated (Figure 2A), and some solid bridges were formed between individual microcapsules. High viscosity not only prevents well-defined particle formation but also tends to form irregular clumps. The microcapsule micrographs obtained show also that indentation and roughness of the surface was more prevalent in smaller particles than in larger ones suggesting that solidification of the walls happened prior to expansion of the microcapsules. The internal structure of the obtained microcapsules show a thicker shell matrix and an air bubble (i.e., void) at the center of the microcapsule (Figure 2B).

### Determination of Minimum Inhibitory Concentrations

In order to compare the antimicrobial activity of limonene, RuL3, and the microencapsulated RuL3, their minimum inhibitory concentrations were measured against *Escherichia coli. Staphylococcus aureus*, *Listeria monocytogenes*, and *Enterococcus faecalis* (Table 1). DMSO (2%) used to enhance the water miscibility of limonene, did not affect the growth of the considered strains at this DMSO concentration (data not shown). The MIC values for limonene against all considered strains were 12.5 mg/ml. Compared with limonene, the MICs (0.4 mg/ml) of the RuL3 complex was approximately 30-fold lower. Thus, the complexation of limonene with 233 Ruthenium enhanced its antibacterial potential. In addition, Table 1 shows that the MIC values of microencapsulated RuL3 were fourfold lower against all studied strains than those of free RuL3. These results clearly show that the Ru–limonene complexation and microencapsulation led to the improvement of the antibacterial efficacy of limonene.

**TABLE 1:** Minimum inhibitory concentration values (mg/ml) of limonene, RuL3, and microencapsulated RuL3 against *Escherichia coli*, *Staphylococcus aureus*, *Listeria monocytogenes*, and *Enterococcus faecalis*.

	MIC mg/ml			
	*E. coli*	*S. aureus*	*L. monocytogenes*	*E. faecalis*
Limonene	12.5	12.5	12.5	12.5
RuL3	0.4	0.4	0.4	0.8
Microencapsulated RuL3	0.1	0.1	0.1	0.4

### Antibiofilm Activity of Studied Compounds

**FIGURE 3:**
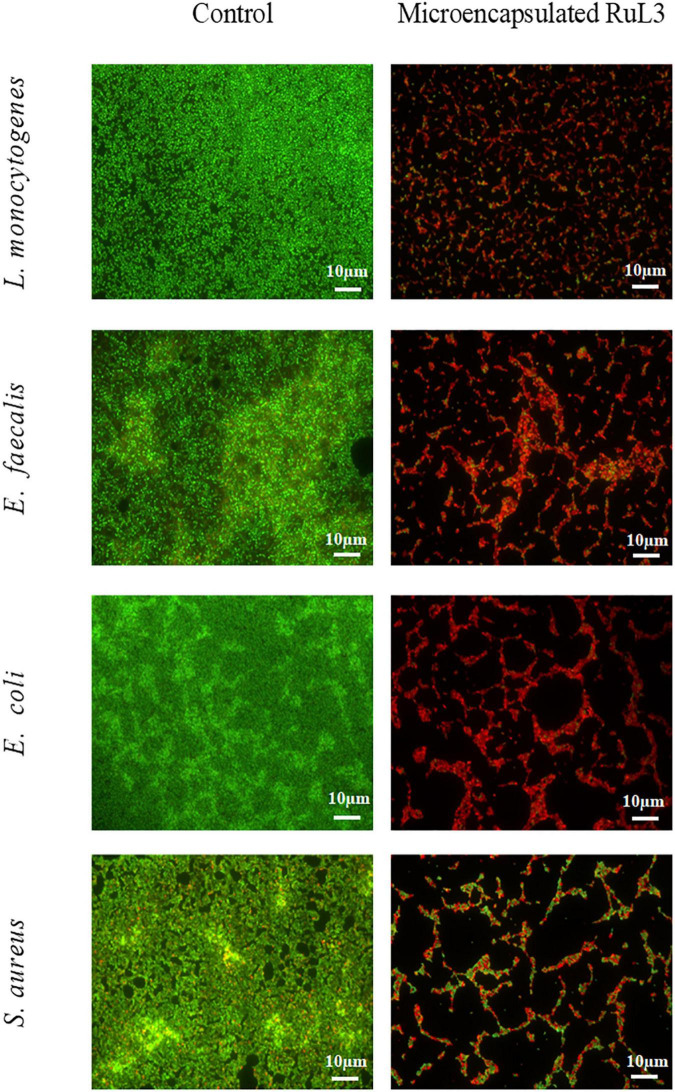
Fluorescence microscope images of *Listeria monocytogenes*, *Enterococcus faecalis*, *Escherichia coli*, and *Staphylococcus aureus* biofilm cells grown at 37°C for 24 h on stainless steel after treatment with microencapsulated RuL3 at the minimum inhibitory concentration (MIC). Cells were visualized after staining with SYTO-9 (green fluorescence for living bacteria) and propidium iodide (red fluorescence for dead bacteria). The control represents cells treated with tryptone salt buffer.

The antibiofilm efficacy of limonene, free and microencapsulated RuL3 were investigated on *E. coli*, *S. aureus*, *L. monocytogenes*, and *E. faecalis* biofilms (Table 2). The results show that treatment with limonene slightly reduced (*p* > 0.05) the initial population of *E. coli*, *S. aureus*, *L. monocytogenes*, and *E. faecalis* biofilms (Table 2). This reduction did not exceed the 0.6 log CFU/cm^2^ regardless of the considered strain (Table 2). However, the RuL3 treatment reduced the initial cell counts of *E. coli*, *S. aureus*, *L. monocytogenes*, and *E. faecalis*, respectively, to 5.5, 5.0, 4.8, and 5.4 log CFU/cm^2^ (Table 2). Moreover, our results show that the treatment with microencapsulated RuL3 led to a reduction in the initial biomass of *E. coli*, *S. aureus*, *L. monocytogenes*, and *E. faecalis*, respectively, to 3.7, 2.8, 3.0, and 4.2 log CFU/cm^2^. This reduction represents reduction of *ca* 90% of the biofilm biomass (Table 2). The log reduction effect of microencapsulated RuL3 was *ca* 2 log higher than that of free RuL3 for *E. coli*, *S. aureus*, and *L. monocytogenes*. However, this difference between free and microencapsulated RuL3 was 1.2 log for *E. faecalis* biofilm. The direct effect of the microencapsulated Rul3 on biofilms (Figure 3) was assessed by using epifluorescence microscopy on 24-h biofilm age of *L. monocytogenes*, *E. faecalis*, *E. coli*, and *S. aureus*. The staining was performed with SYTO9 and PI after treatment with microencapsulated RuL3. Biofilms treated with TS (control) show a dense and covering layer of viable biomass mainly stained with SYTO9. Treatment with RuL3 microcapsules resulted in a significant dispersion of biofilm biomass from the surface of SS coupon (Figure 3). Furthermore, the majority of the cells in the remaining biofilm was stained with PI, reflecting the significant biofilm elimination action of the proposed RuL3 microcapsules.

**TABLE 2:** *Escherichia coli*, *Staphylococcus aureus*, *Listeria monocytogenes*, and *Enterococcus faecalis* biofilm log counts after treatment for 10 min with limonene, free and microencapsulated RuL3 at their respective MICs.

	*E. coli*	*S. aureus*	*L. monocytogenes*	*E. faecalis*
**Log CFU/cm^2^**				
Control	8.1 ± 0.3	8.3 ± 0.2	7.8 ± 0.2	7.4 ± 0.3
Limonene	7.5 ± 0.1	7.7 ± 0.2	7.3 ± 0.3	6.9 ± 0.1
RuL3	5.5 ± 0.2	5.0 ± 0.2	4.8 ± 0.3	5.4 ± 0.1
Microen- capsulated RuL3	3.7 ± 0.3	2.8 ± 0.1	3.0 ± 0.1	4.2 ± 0.2

*For the control, biofilms were treated with potassium phosphate buffer (100 mM, pH 7) for 10 min.*

### Impact of Free and Microencapsulated RuL3 Treatment on *E. coli* Morphology

**FIGURE 4:**
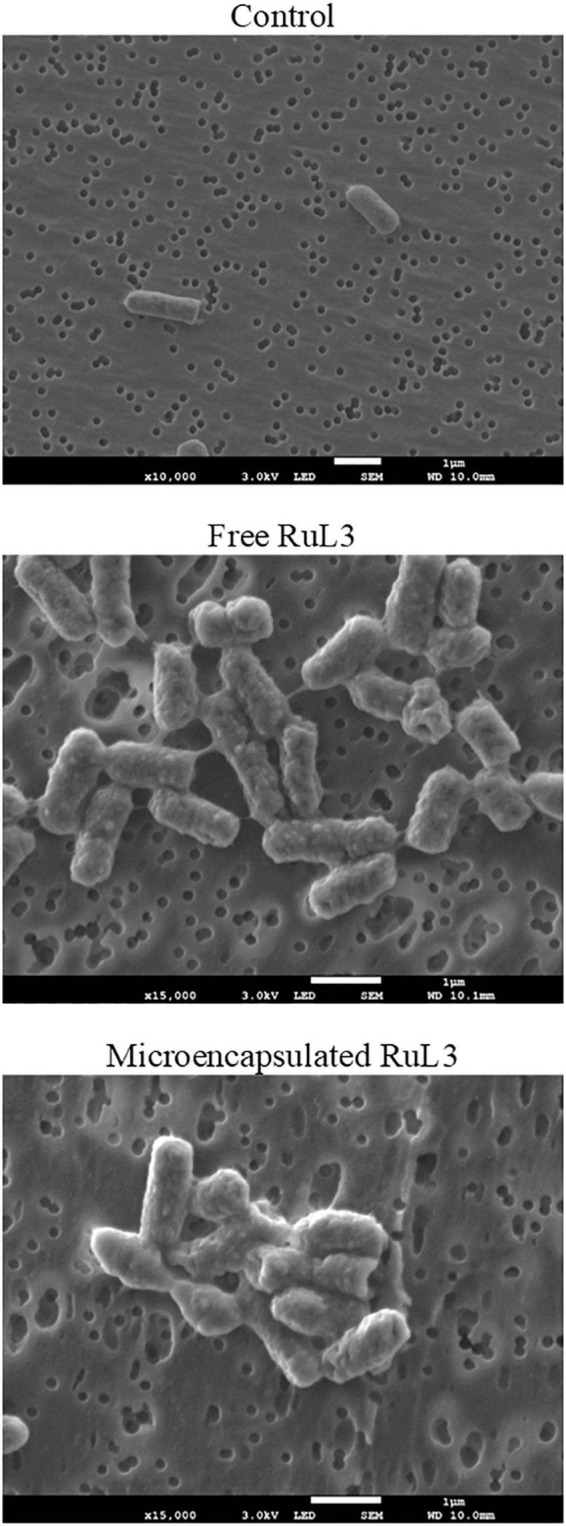
Scanning electron micrographs of *Escherichia coli* biofilm cells, after treatment with free and micoencapsulated RuL3 at the MIC. The control represents cells treated with potassium phosphate buffer.

*Escherichia coli* was used in order to measure the effect of free and microencapsulated RuL3 treatments on the bacterial cells. The bacterial cells were exposed to the MIC values of free and microencapsulated RuL3. The structural morphology of the treated *E. coli* was observed by SEM (Figure 4). Figure 4 shows that untreated cells (control) had a regular and normal bacilliform structure. However, the micrographs of the cells treated with free and microencapsulated RuL3 shows different forms of distortion and deformation (Figure 4). Furthermore, treated cells endured a complete collapse of the morphology of the cells accompanied by intercellular pool leakage (Figure 4). Similar results were obtained with *Staphylococcus aureus* (data not shown). These results provide evidence that the microencapsulated RuL3 was endowed with a stronger antimicrobial activity against the *E. coli* compared with its free form.

### *Escherichia coli* Cytoplasmic Membrane Permeabilization by Free and Microencapsulated RuL3

**FIGURE 5:**
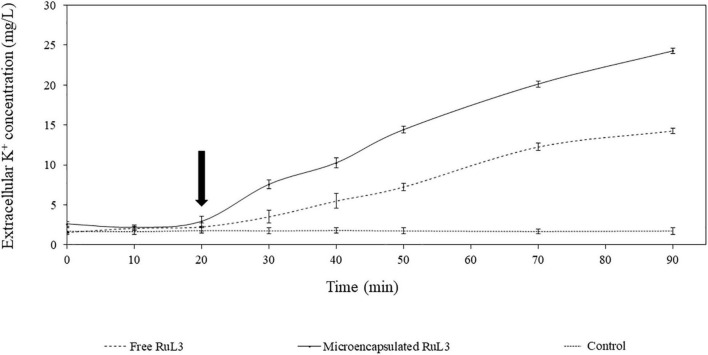
Kinetics of intracellular potassium leakage from *Escherichia coli* cells after treatment with either free or microencapsulated RuL3 at the minimum inhibitory concentration. The K^+^ concentration is presented in milligrams per liter. The treatment with TSB prepared in 50 mM MOPS buffer represents the negative control. The arrow indicates when free or microencapsulated RuL3 was added. Data are presented as means (± SD) of three independent repeats in triplicate.

The effect of free and microencapsulated RuL3 on the permeability of *E. coli* membranes was investigated (Figure 5). In this context, the leakage of intracellular K^+^ ions was monitored. Our results show that the addition of TSB (prepared in 50 mM MOPS buffer) had no effect on K^+^ efflux, which remained stable (control). However, when *E. coli* cells were exposed to the free (0.4 mg/ml) and microencapsulated RuL3 (0.1 mg/ml) (final concentration corresponding to the MIC), the result was an immediate and rapid increase in K^+^ concentration in the suspension medium. Seventy minutes after exposing cells to free and microencapsulated RuL3, the K^+^ concentration in the suspension medium increased to 14 and 24 mg/L, respectively (Figure 5). These results show that the microencapsulated RuL3 (0.1 mg/ml) was more efficient than the free RuL3 (0.4 mg/ml) even at a concentration that was four times lower.

**FIGURE 6:**
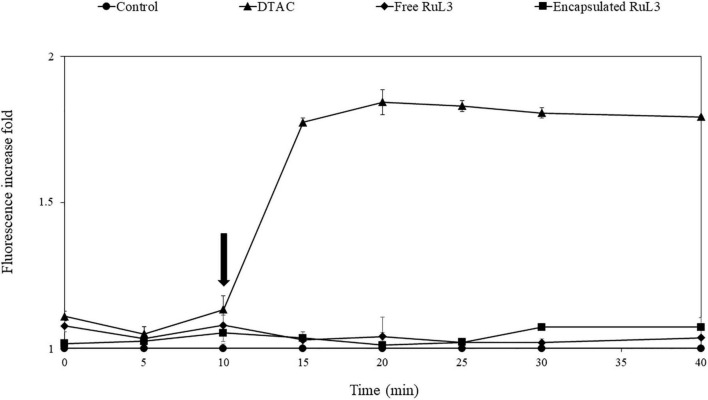
Assessment of *Escherichia coli* (ATCC 25922GFP) cytoplasmic membrane permeability when exposed to dodecyltrimethylammonium chloride (DTAC), free RuL3, or encapsulated RuL3 at the minimal inhibitory concentration. The black arrow indicates the time at which (DTAC) free RuL3 or encapsulated RuL3 was added. Fluorescence of the green fluorescent protein was measured spectroscopically at 485- to 510-nm excitation and emission wavelengths. Data are presented as means (± SD) of three independent repeats in triplicate. The control represents cells treated with HEPES buffer.

To check out if the membrane damage leads to intracellular protein leakage, an *E. coli* GFP strain was used to monitor extracellular GFP fluorescent intensity after the exposure of the bacterial cells grown at 37°C to free and microencapsulated RUL3. Dodecyltrimethylammonium chloride (DTAC) was used as the positive control. The results showed no significant effect on the fluorescence intensity (Figure 6) after the addition of TSB (prepared in 50 mM MOPS buffer) as a negative control. After the exposure of bacterial cells to DTAC, an instantaneous and significant increase in the extracellular fluorescence intensity was measured (Figure 6). After 10 min, the GFP fluorescence intensity increased by 1.5-fold in the supernatant filtrates of the bacterial suspension treated with DTAC. However, no fluorescence increase was measured after the addition of the free microencapsulated RUL3. These results were in agreement with these obtained by an analysis, using SDS page electrophoresis, of intracellular proteins in the supernatant before and after the addition of the free or microencapsulated RUL3 (data not shown). These results show that the free or microencapsulated RUL3 induce potassium release but not protein leakage from the cytoplasm.

### Cytotoxicity of Free and Microencapsulated RuL3

**FIGURE 7:**
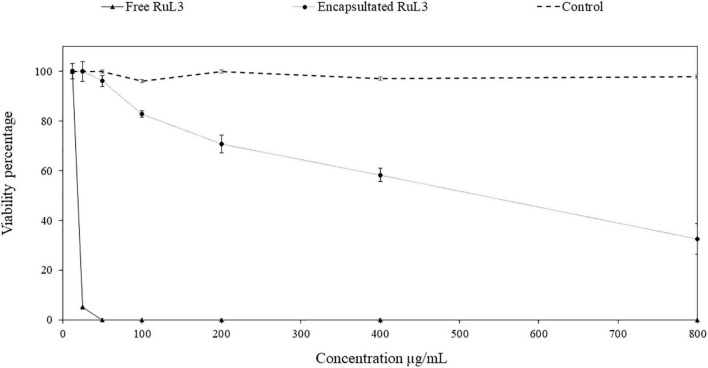
*HeLa* cell viability percentage after 24-h exposure to concentrations (between 12.5 and 800 μg/ml) of free and microencapsulated RuL3. The control represents the spray-dried microcapsules without RuL3 (empty capsules). Data are presented as means (± SD) of three independent repeats in triplicate.

The viability of *HeLa* cells was assessed after exposure for 24 h to free or microencapsulated RuL3 at twofold serial dilutions. The final concentration ranged from 12.5 to 800 μg/ml (Figure 7). The DMEM with 10% FBS was used as a negative control. An additional control was used consisting of microcapsules formulated without RuL3. The treatment with empty capsules had no cytotoxic effect as the viability of *HeLa* cells remained at *ca* 100% regardless of the considered concentration (Figure 7). Contact with 12.5 μg/ml of free RuL3 shows no negative effect on *HeLa* cell viability, which remained at 100% (Figure 7). However, when incubated in the presence of 25 μg/ml of free RuL3, the viability percentage was significantly (*p* < 0.05) reduced to 5% (Figure 7). At concentrations ≥ 50 μg/ml, the viability percentage was reduced to 0% (Figure 7). Figure 7 shows that microencapsulated RuL3 had lower cytotoxicity toward *HeLa* cells compared with its free form (*p* < 0.05). At a concentration of 50 μg/ml, the cell viability was of 96% (*p* < 0.05). *HeLa* cell viability was reduced to a mean value of 32% in contact with 800 μg/ml of encapsulated RuL3.

## Discussion

Ruthenium complexes (Ru), initially developed for anticancer treatment purposes (Benabdelaouahab et al., 2016), have recently attracted interest as a new class of antimicrobial agents (Li et al., 2015; Southam et al., 2017; Yang et al., 2018). Moreover, ruthenium complexes are being pursued to help overcome the multidrug resistance bacteria (Claudel et al., 2020; Di Natale et al., 2020).

This study explored the antibacterial and the antibiofilm activity of free and microencapsulated RuL3 compared with limonene against four foodborne pathogenic bacteria. The RuL3 was synthesized, and its zeta potential was studied as a function of pH. The point at which the overall charge of RuL3 was neutral was between 5.5 and 6 as shown in our study. The information provided by this result was important because the antimicrobial activity depends on the interactions between the active molecule and the membrane of the target microorganisms, which, in turn, is a function of the electric charge of the molecule. Thus, we worked at pH 7, a condition in which the molecule had an overall negative charge, which can play an important role in the intensity of its antimicrobial activity (Khelissa et al., 2021a,b).

The MIC values indicated that limonene had an inhibitory activity against *E. coli*, *S. aureus*, *L. monocytogenes*, and *E. faecalis*. However, the MICs of limonene dissolved in DMSO has shown a very high value of *ca* 12.5 mg/ml. Moreover, our results show that free RuL3 significantly inhibits the growth of *E. coli*, *S. aureus*, *L. monocytogenes*, and *E. faecalis* at a concentration up to 30-fold lower than those of limonene (Table 1). The microencapsulation of RuL3 reduced the initial MIC of free RuL3 by fourfold against *E. coli*, *S. aureus*, and *L. monocytogenes* and by twofold against *E. faecalis*. Among the studied bacteria, the highest MICs were observed against *E. faecalis* whatever the tested compound is. These results are consistent with our previous studies, which show that the microencapsulation of different disinfectants may enhance their antibacterial activity against several food pathogens (Khelissa et al., 2021a,b). Furthermore, the most interesting feature was that the microencapsulated RuL3 antibacterial activity against *E. coli*, *S. aureus*, *L. monocytogenes*, and *E. faecalis* biofilm cells was significantly (*p* < 0.05) higher than that of free RuL3. These results are in agreement with several studies reporting that the structure of the antimicrobial agent influences its interactions with biofilm matrix components and, thus, its disinfection efficacy (Singh et al., 2017; Karygianni et al., 2020).

The SEM micrographs clearly show that both free and microencapsulated RuL3 were able to damage the morphology of *E. coli*. The destruction of cell wall and cell membrane could lead to an irreversible leakage of the intracellular pool, resulting in cell death. In fact, the bacterial cell membrane acts as a natural protective barrier against many antimicrobials (Claessen and Errington, 2019). Exposure of bacteria to microencapsulated RuL3 resulted in significant biofilm dispersal and an increase in cell death. The results are consistent with our previous studies conducted on microencapsulated quaternary ammonium compounds (Khelissa et al., 2021a,b). When cell membranes of bacteria are destroyed, after treatment with disinfectant, the internal electrolyte leaks into the culture medium (Han et al., 2019). It is known that under normal physiological conditions, the intracellular concentration of K^+^ is higher than that in the extracellular medium (Stratford et al., 2019). In this study, the exposure of *E. coli* to RuL3 under its free and microencapsulated forms was observed to significantly increase the extracellular K^+^ concentration, which reflects the permeabilization of the cell membrane and the irreversible leakage of bacterial intracellular pool. Nevertheless, the final extracellular concentration of K^+^ after *E. coli* treatment with microencapsulated RuL3, was 1.7-fold higher than that measured after treatment with free RuL3. Thus, the results underlined that microencapsulation enhances the antibacterial action of RuL3. These finding are in agreement with our previous study reporting that microencapsulation of DTAC significantly enhanced its membrane-disturbing action compared with free DTAC (Khelissa et al., 2021b). Taken together, the results show that RuL3 is likely to target the bacterial cytoplasmic membrane and disturb the integrity of its phospholipidic bilayer. These results are consistent with the results of SEM observation insofar as they highlighted the bacterial membrane collapse. In addition, these results are in agreement with those from our previous studies reporting that spray-drying microencapsulation can be used as an effective tool to enhance the activity of the antibacterial agent while reducing the required concentration and the associated cytotoxicity (Khelissa et al., 2021a,b). Our finding also showed that the free or microencapsulated RUL3 induced no protein leakage from the cytoplasm in contrast to DTAC, which, when added to *E. coli* GFP resulted in a leakage of intracellular proteins as reported in our previous results (Khelissa et al., 2021b).

Ruthenium complexes are toxic and selective toward cancer cells as reported (Benabdelaouahab et al., 2016). They have exhibited low *in vitro* toxicity associated with a good *in vivo* antimetastatic characteristics (Scolaro et al., 2005). Our results are in agreement with these reports as, at a concentration of 25 μg/ml, the free RuL3 reduced cell viability by 95% of *HeLa* cells (Figure 7). The current results show that the microencapsulation of RuL3 significantly reduced its cytotoxicity toward *HeLa* cells. These results show that biosourced terpenes could be used to synthetize complexes, such as RuL3, which have a great antibacterial and antibiofilm activity. The encapsulation of the RuL3 appears as a promising approach, to reduce the amount of the antimicrobial used and enhancing at the same time its efficiency toward biofilm and biofouling. The encapsulation of RuL3 allows reducing its cytotoxicity. This will help to set up an efficient disinfection strategy with less negative environmental impact. In addition, our original study reports the successful synthesis and microencapsulation of RuL3 complex using spray-drying process. Additionally, results illustrate that the microencapsulation of the RuL3 complex improves its efficiency against biofilms of food pathogenic bacteria and promotes a significant decrease in its cytotoxicity. The RuL3 has likely targeted the bacterial cytoplasmic membrane and promoted its irreversible collapse. Thus, these important feature makes this ruthenium complex an interesting alternative for the conventional range of commercialized antibacterial agents.

## Data Availability Statement

The original contributions presented in the study are included in the article/supplementary material, further inquiries can be directed to the corresponding author/s.

## Author Contributions

SK contributed to the conception and design of the study and manuscript redaction and performed the microbiology experiments. YF and M-AA performed the chemistry experiments and contributed to manuscript redaction. SM performed the membrane damage experiments using Escherichia coli GFP strain. SA contributed to manuscript writing and reviewing its final version. AG performed the microencapsulation experiments and contributed to the process of writing of the manuscript. AB performed the cytotoxicity assays. N-EC supervised the work, designed the study, and contributed to the redaction and revision of the manuscript. All authors approved the submitted version.

## Conflict of Interest

The authors declare that the research was conducted in the absence of any commercial or financial relationships that could be construed as a potential conflict of interest.

## Publisher’s Note

All claims expressed in this article are solely those of the authors and do not necessarily represent those of their affiliated organizations, or those of the publisher, the editors and the reviewers. Any product that may be evaluated in this article, or claim that may be made by its manufacturer, is not guaranteed or endorsed by the publisher.
